# EGFR Interacts with the Fusion Protein of Respiratory Syncytial Virus Strain 2-20 and Mediates Infection and Mucin Expression

**DOI:** 10.1371/journal.ppat.1005622

**Published:** 2016-05-06

**Authors:** Michael G. Currier, Sujin Lee, Christopher C. Stobart, Anne L. Hotard, Remi Villenave, Jia Meng, Carla D. Pretto, Michael D. Shields, Minh Trang Nguyen, Sean O. Todd, Michael H. Chi, Jason Hammonds, Stefanie A. Krumm, Paul Spearman, Richard K. Plemper, Kaori Sakamoto, R. Stokes Peebles, Ultan F. Power, Martin L. Moore

**Affiliations:** 1 Department of Pediatrics, Emory University, Atlanta, Georgia, United States of America; 2 Children’s Healthcare of Atlanta, Atlanta, Georgia, United States of America; 3 Centre for Infection and Immunity, School of Medicine, Dentistry and Biomedical Science, Queens University Belfast, Belfast, Northern Ireland; 4 The Royal Belfast Hospital for Sick Children, Belfast, Northern Ireland; 5 Division of Allergy, Pulmonary, and Critical Care Medicine, Vanderbilt University School of Medicine, Nashville, Tennessee, United States of America; 6 Institute for Biomedical Sciences, Georgia State University, Atlanta, Georgia, United States of America; 7 Department of Pathology, College of Veterinary Medicine, University of Georgia, Athens, Georgia, United States of America; University of North Carolina at Chapel Hill, UNITED STATES

## Abstract

Respiratory syncytial virus (RSV) is the major cause of viral lower respiratory tract illness in children. In contrast to the RSV prototypic strain A2, clinical isolate RSV 2–20 induces airway mucin expression in mice, a clinically relevant phenotype dependent on the fusion (F) protein of the RSV strain. Epidermal growth factor receptor (EGFR) plays a role in airway mucin expression in other systems; therefore, we hypothesized that the RSV 2–20 F protein stimulates EGFR signaling. Infection of cells with chimeric strains RSV A2-2-20F and A2-2-20GF or over-expression of 2–20 F protein resulted in greater phosphorylation of EGFR than infection with RSV A2 or over-expression of A2 F, respectively. Chemical inhibition of EGFR signaling or knockdown of EGFR resulted in diminished infectivity of RSV A2-2-20F but not RSV A2. Over-expression of EGFR enhanced the fusion activity of 2–20 F protein in *trans*. EGFR co-immunoprecipitated most efficiently with RSV F proteins derived from “mucogenic” strains. RSV 2–20 F and EGFR co-localized in H292 cells, and A2-2-20GF-induced MUC5AC expression was ablated by EGFR inhibitors in these cells. Treatment of BALB/c mice with the EGFR inhibitor erlotinib significantly reduced the amount of RSV A2-2-20F-induced airway mucin expression. Our results demonstrate that RSV F interacts with EGFR in a strain-specific manner, EGFR is a co-factor for infection, and EGFR plays a role in RSV-induced mucin expression, suggesting EGFR is a potential target for RSV disease.

## Author Summary

Respiratory syncytial virus (RSV) is responsible for severe lower respiratory disease in infants and young children. Overabundant airway mucus contributes to airway obstruction in RSV bronchiolitis, and a better understanding of RSV pathogenesis may contribute to needed therapies and vaccines. We reported previously that RSV clinical isolate strain 2–20 induces more airway mucin expression in mice than prototypic RSV strains and that the 2–20 fusion (F) protein mediates mucin induction. Epidermal growth factor receptor (EGFR) has been shown to play a role in lung mucin expression. We identified a functional interaction between 2–20 F and EGFR, in that 2–20 F expression activated EGFR and, reciprocally, EGFR expression increased 2–20 F fusion activity. RSV F and EGFR co-localized in infected cells. EGFR co-immunoprecipitated with RSV F protein from various RSV strains, and the strength of this *in vitro* interaction correlated with strain-specific airway pathogenicity in mice. EGFR inhibition abrogated 2–20 F-mediated infection *in vitro* and mucin expression induction *in vivo*. These data identify EGFR as a novel strain-specific co-factor of RSV infection and suggest EGFR may be a target for ameliorating RSV disease.

## Introduction

Respiratory syncytial virus (RSV) is a human pathogen of the *Pneumovirus* genus within the *Paramyxoviridae* family. Worldwide, the virus causes over 30 million lower respiratory tract illnesses per year in children and is a leading cause of infant pneumonia mortality [1, 2]. Despite a substantial clinical burden of disease, there are no available vaccines or RSV-specific therapeutics. A challenge to RSV vaccine and therapy strategies remains elucidation of the unclear relationship between RSV infection and pathogenesis.

RSV is an enveloped, non-segmented, negative-strand RNA virus whose genome is approximately 15.2 kb in length and encodes 10 genes which are translated into 11 proteins. RSV attachment is mediated through host glycosaminoglycans (GAGs), cellular protein nucleolin, association with cholesterol-rich microdomains, and CX3CR1 [3–9]. Mechanisms surrounding RSV entry remain unclear and other host receptors, co-receptors, and co-factors contributing to infection are likely to be identified. Two envelope proteins mediate RSV infection, the attachment glycoprotein (G) and the fusion (F) protein. Prior to infection, RSV F exists in a metastable pre-fusion conformation [10, 11]. RSV F undergoes a series of conformational changes yielding a thermodynamically stable six-helix post-fusion bundle, which drives viral and host membrane fusion [11–13]. RSV G is mucin-like, having extensive N- and O-linked glycosylation, and G is responsible for facilitating RSV attachment through interactions with GAGs and CX3CR1 [4, 6, 7, 9, 14, 15]. However, G is not absolutely required for viral entry into immortalized monolayer cells [16–18]. Mechanisms by which F and G mediate host cell entry and their interactions with other host cell targets remain uncertain.

Epidermal growth factor receptor (EGFR) is a host glycoprotein comprised of an extracellular ligand receptor and intracellular kinase domain. The latter is activated through both Src-dependent phosphorylation and autophosphorylation [19, 20]. In addition to a wide variety of host ligands including epidermal growth factor (EGF) and transforming growth factor alpha (TGFα), several viruses have been identified that employ EGFR binding and activation for viral entry and replication. These pathogens include hepatitis B virus, human cytomegalovirus (hCMV), and Epstein-Barr virus (EBV) [21–23]. Previous studies by others evaluating the role of EGFR in RSV infection have shown that RSV activates EGFR in lung epithelial cells [24, 25]. EGFR activation in these cells promotes a pro-inflammatory response including increased survival of RSV-infected cells and suppression of interferon regulatory factor (IRF) 1-dependent CXCL10 production, an important event for recruitment of lymphocytes to infected airway epithelial cells [24, 25]. Another study using a recombinant virus based on the RSV subgroup A prototypic strain A2 demonstrated that RSV cell entry is largely mediated through endocytotic macropinocytosis promoted by EGFR phosphorylation [26].

Respiratory failure is the critical consequence of RSV disease in children, and overabundant mucus obstruction of the airways contributes to this outcome. Our laboratory previously reported that clinical isolate RSV A2001/2-20 (2–20) causes more airway necrosis, inflammation, and mucin expression during infection in BALB/cJ mice than the A2 reference strain [27, 28]. Transfer of the RSV 2–20 F protein into strain A2 recapitulated higher levels of airway mucin expression in mice [28]. These studies demonstrated that the RSV F protein plays a key role in airway epithelium infection and pathogenesis *in vivo* and suggests that RSV F plays a role in RSV strain-specific phenotypes. EGFR phosphorylation is known to play a role in mucin expression in airway epithelial cells during influenza and rhinovirus infections [29, 30]. We hypothesized that mucin induction by RSV 2–20 F is mediated by a specific interaction with EGFR. To test this hypothesis, we evaluated the ability of A2 and 2–20 viruses and transiently expressed F proteins to activate EGFR, and we assessed the impact of disrupting these interactions on virus infectivity *in vitro* and mucin expression *in vivo*. We demonstrate that RSV 2–20 F protein specifically binds to and activates EGFR, EGFR contributes to RSV-2-20 F infectivity, and EGFR signaling mediates 2–20 F induction of airway mucin expression in mice.

## Results

### RSV 2–20 F activates EGFR, and EGFR promotes 2–20 F protein activity

**Fig 1:**
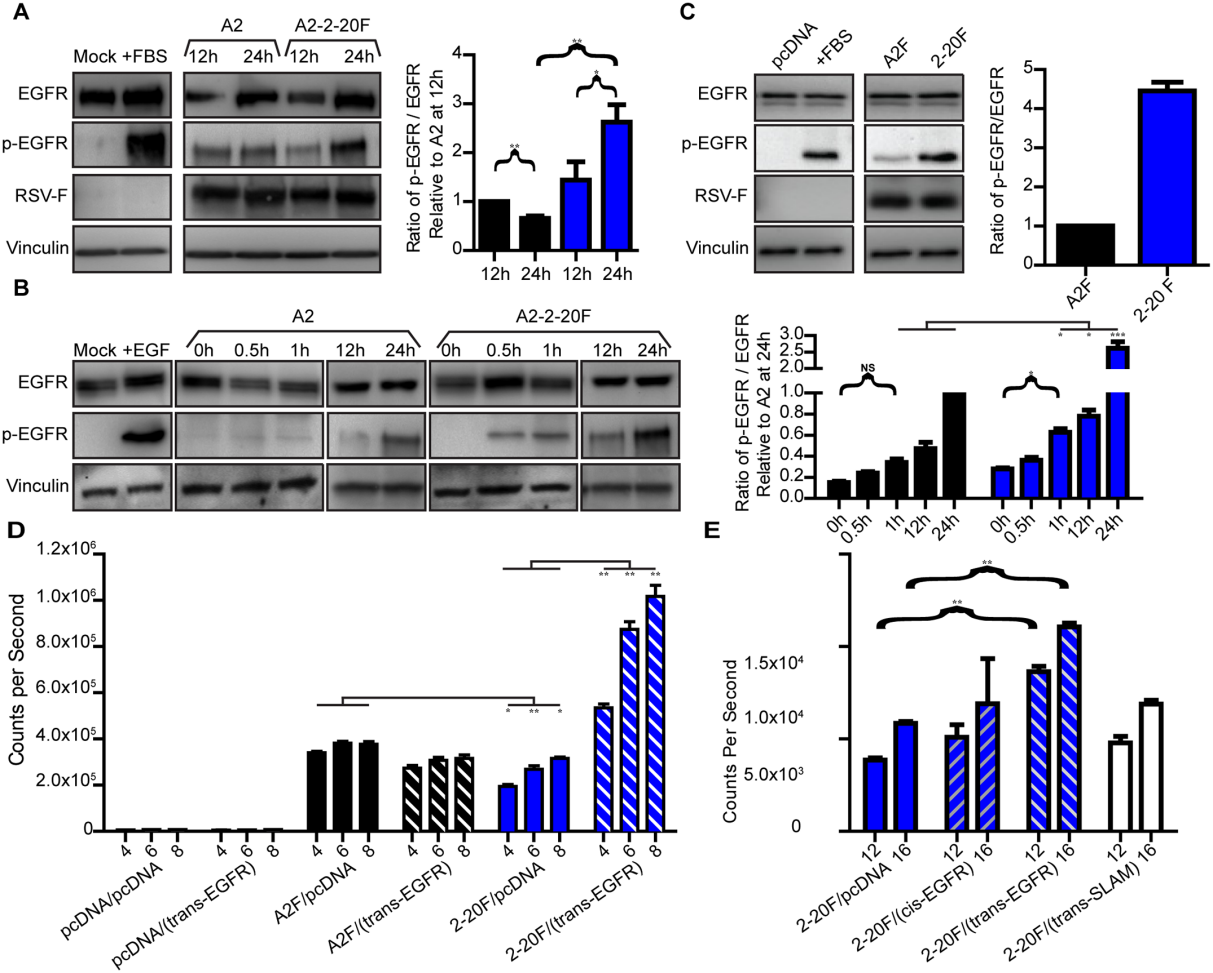
RSV 2–20 F activates EGFR and vice versa. **(A)** Representative total EGFR and p-EGFR Western blots from serum-starved HEp-2 cells 12 and 24 hr after infection at an MOI = 3 with RSV A2 or A2-2-20F. Mock infection and FBS were controls. Densitometry of four experimental replicates providing the ratio of detected p-EGFR to EGFR is shown below. (**B**) Total and p-EGFR levels from serum-starved H292 cells 0, 0.5, 1, 12, and 24 hr after infection at MOI = 3. (**C**) Representative EGFR and p-EGFR Western blots from serum-starved HEp-2 cells 24 h after transfection with vectors expressing A2 F or 2–20 F protein. Quantification of the ratio of detected p-EGFR to EGFR from three experiments is shown at right. (**D**) Cell-cell fusion activity was quantified by the DSP assay as described in the text and Materials and Methods. Cell-cell fusion was measured 4, 6, and 8 h after mixing effector and target cells. (**E**) Cell-cell fusion 12 and 16 h after cell mixing following transfection with EGFR in either the same effector cells (*cis*) as 2–20 F or in the target (*trans*) cells. Error bars represent standard error of the mean (SEM). * and ** represent significant differences (*P* < 0.05 and *P* < 0.005, respectively by ANOVA). Graphs in (**C**) and (**D**) depict three experimental replicates combined.

As EGFR phosphorylation/activation mediates mucin expression in response to other respiratory viruses, RSV A2 is known to activate EGFR, and RSV 2–20 F is more “mucogenic” than A2 F in the context of infection, we hypothesized that the 2–20 strain F protein potently activates EGFR. Western blotting was performed to determine levels of total EGFR and phospho-EGFR (p-EGFR) after infection of HEp-2 cells with RSV strains A2 and A2-2-20F. We used serum-starved cells because serum is an activator of EGFR signaling in vitro [31, 32]. A2-2-20F-infected cells had a higher ratio of p-EGFR to EGFR than A2-infected cells at 24 h post-infection (Fig 1A). We performed similar experiments in serum-starved NCI-H292 (H292) cells, and in an earlier time course we found that A2-2-20F infection resulted in a higher p-EGFR/EGFR ratio than A2 infection at 1, 12, and 24 hr post-infection (Fig 1B). To define the role of RSV 2–20 F expression alone in EGFR activation, A2 F and 2–20 F expression constructs were transfected into serum-starved HEp-2 cells and blotted for EGFR and p-EGFR levels. There was a significantly higher 4.45-fold ratio of p-EGFR to EGFR after 2-20F expression than after A2 F expression (Fig 1C). These data demonstrate that the 2–20 RSV F protein activated EGFR in cells to a greater extent than the A2 strain F protein.

We tested whether EGFR can enhance RSV F protein fusion activity. We used a previously established cell-cell fusion assay [28, 33, 34]. The dual-split protein (DSP) assay is based on co-transfecting an “effector”/*cis* population of 293T cells with a construct expressing N-terminal domains of a *Renilla* luciferase and GFP fusion protein (DSP_1-7_) and an F expression construct in the presence of specific fusion inhibitor. Another population of 293T cells (“target”/*trans*) is transfected with a construct expressing the C-terminal domains of the luciferase-GFP fusion protein (DSP_8-11_) and, in this case, either equal molar amounts of empty vector (pcDNA) or an EGFR expression vector. The fusion inhibitor is washed out, the effector and target cells are mixed, and fusion is quantified by luciferase activity reconstituted by cell content mixing. A2 F had significantly more cell-cell fusion activity than 2-20F in this assay (Fig 1D). Expression of EGFR in *trans* enhanced 2–20 F activity but not A2 F activity (Fig 1D). Similar to published A2 F and 2–20 F fusion assay experiments [28], we found no difference, as measured by flow cytometry, between A2 F and 2–20 F surface expression in 293T cells (S1 Fig). To determine whether the boost in RSV 2-20F fusion is specific to a *trans* F-EGFR interaction, 2-20F was either co-expressed (in *cis*) with EGFR or in *trans* in the target cells. There was a significant boost to 2-20F fusion when EGFR was expressed in *trans* but not in *cis*, suggesting EGFR enhancement of 2–20 F activity does not occur when the proteins are overexpressed in the same cells or membrane (Fig 1E). Co-expression of EGFR with RSV F did not alter F surface expression (S1 Fig). There was no effect on 2–20 F activity by overexpression of signaling lymphocyte activation molecules (SLAM), a receptor for measles virus (MeV) serving as an irrelevant transmembrane protein control. Collectively, the data show EGFR specifically enhanced the fusion activity of RSV strain 2–20 F protein but not A2 F protein.

### EGFR inhibition and knockdown reduced infectivity of RSV expressing 2–20 F

**Fig 2:**
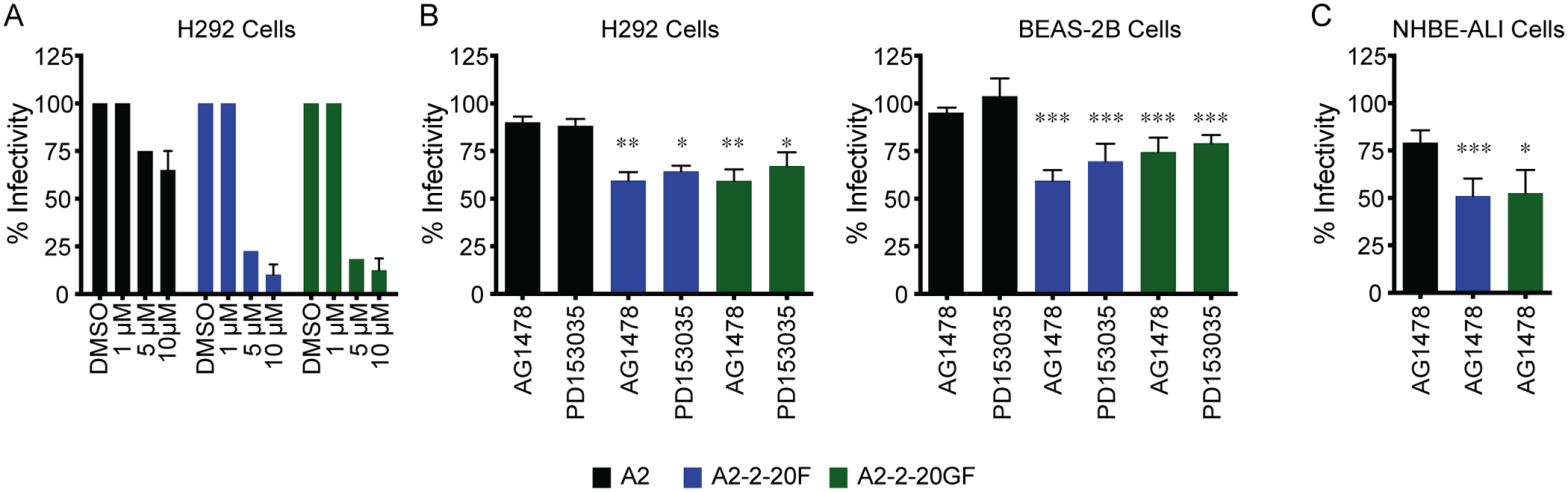
EGFR Inhibition Results in Reduced Infectivity of RSV A2-2-20F and A2-2-20GF. (**A**) Serum-starved H292 cells were pre-treated for 1 h with DMSO vehicle or the indicated concentrations of AG1478 prior to infection with RSV A2, A2-2-20F, or RSV A2-2-20GF at an MOI of 1. Following 24 h of infection in the presence of inhibitor, cells were harvested and evaluated for infection by flow cytometry for mKate2 expression. The percent infectivity was normalized to infectivity of the strain in vehicle treatment. (**B**) Serum-starved H292 (left) or BEAS-2B (right) cells were pre-treated with either 10 μM AG1478 or 400 nM PD153035 and infected as described with the indicated RSV strains at MOI = 3. (C) NHBE-ALI cultures were pre-treated with 5 μM AG1478 then infected with the indicated RSV strains at MOI = 3. Error bars represent standard error of the mean (SEM). *, **, and *** represent significant differences (*P* < 0.05, *P*<0.005 and *P* < 0.0005, respectively by ANOVA) between the drug treatment conditions compared to A2 infection. Graphs in (**A**), (**B**), and (C) represent three experimental replicates combined.

EGFR can function as receptor, co-receptor, or entry co-factor for other viruses, and EGFR depletion in HeLa cells was reported to reduce RSV A2 strain infectivity [26]. We explored the role of EGFR in RSV-2-20F infection. Cells were pre-treated with EGFR tyrosine kinase inhibitors (AG1478 and PD153035) then infected with recombinant RSV A2, RSV A2-2-20F, or RSV A2-2-20GF. Virus infectivity was measured by flow cytometry of virally expressed mKate2. NCI-H292 (H292) cells are a human tracheal epithelial cell line known to express mucin genes through activation of the EGFR pathway and known to support RSV replication [35–37]. Treatment of H292 cells with increasing concentrations of EGFR inhibitor AG1478 resulted in a dose-dependent reduction in infectivity of all three virus strains at an MOI of 1, and the reduction in infection was greater against A2-2-20F and A2-2-20GF than against A2 (Fig 2A). Treatment of H292 cells prior to RSV A2 infection at a higher MOI = 3 with either AG1478 or PD153035 resulted in no change in infection (Fig 2B). In contrast, EGFR inhibition prior to A2-2-20F or A2-2-20GF infection at MOI = 3 reduced infection efficiency (Fig 2B). Treatment in BEAS-2B cells, a human bronchial epithelial cell line, with AG1478 or PD153035 resulted in similar decreases in infectivity of RSV A2-2-20F and A2-2-20GF while having no effect on A2 (Fig 2B). In order to test the effect of EGFR inhibition on RSV infectivity in a model more relevant to RSV biology, we utilized normal human bronchial epithelial cells differentiated at air-liquid interface (NHBE-ALI) [38]. We found that pre-treatment of NHBE-ALI cultures with 5 μM AG1478 was not toxic, as previously described, whereas PD153035 was toxic to NHBE-ALI [39]. Pre-treatment of NHBE-ALI cultures with AG1478 resulted in significant reduction in infectivity of RSV A2-2-20F and A2-2-20GF, and no significant effect on A2 compared to vehicle (Fig 2C). Taken together, EGFR signaling mediated infection by RSV expressing the 2–20 F protein.

**Fig 3:**
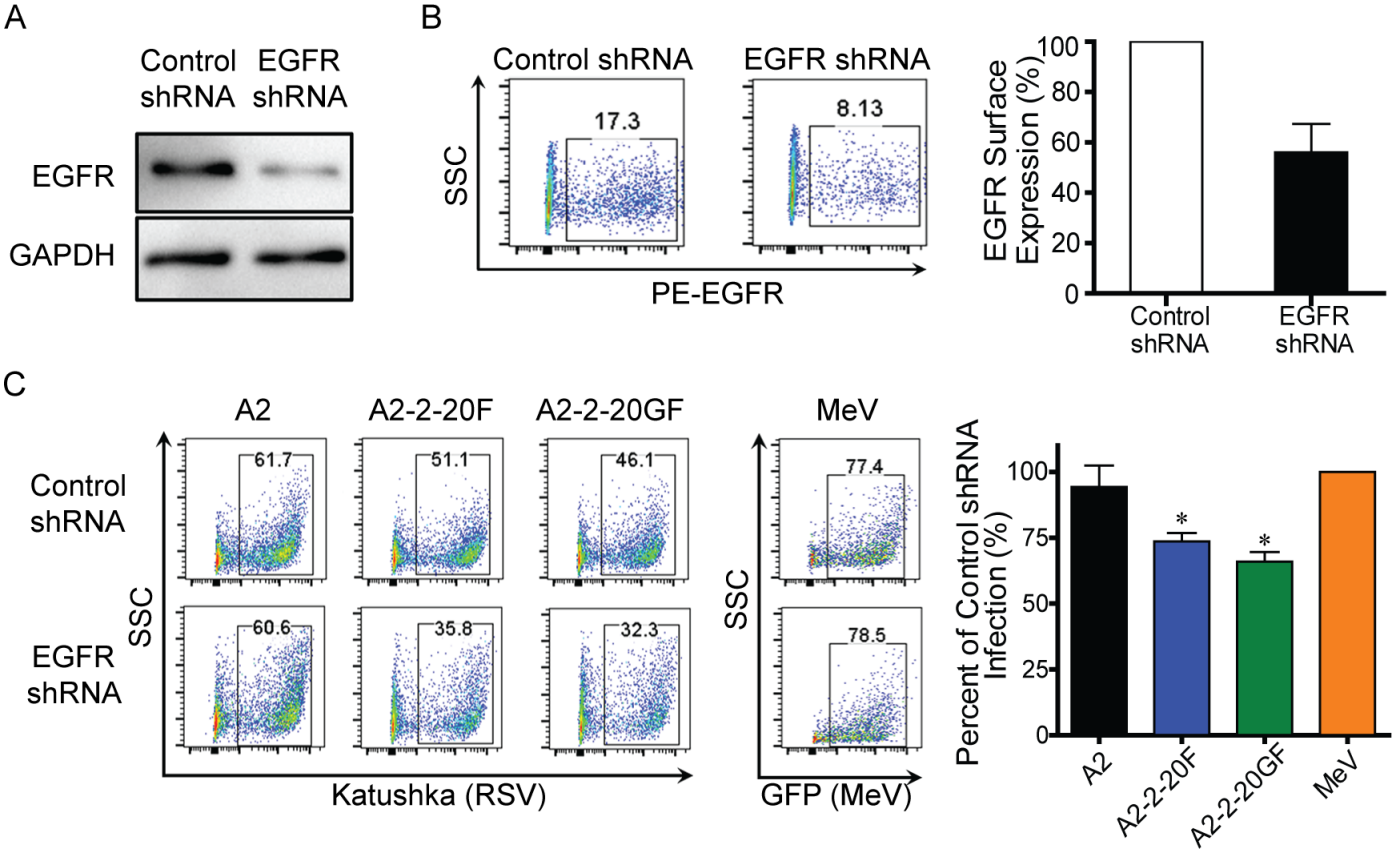
Knockdown of EGFR in BEAS-2B Cells Results in RSV Strain-Specific Reduction of Virus Infection. **(A–B)** BEAS-2B cells were treated with scrambled (Control) shRNA or EGFR-specific shRNA by lentivirus transduction. Representative images of reduction in EGFR expression detected by Western blot (**A**) probing for EGFR and by flow cytometry (**B**) are shown. Quantification of the flow cytometry data from two experimental replicates is shown. (**C**) BEAS-2B cells treated with EGFR-specific shRNA were infected with RSV A2 (black), A2-2-20F (blue), A2-2-GF (green), or GFP-MeV (orange), and infection evaluated by flow cytometry. Representative images are shown on the left and quantification of three experimental replicates on the right. Error bars represent standard error of the mean (SEM) and * represents significant differences (*P* < 0.05 by ANOVA) compared to A2 infection.

To determine the role of EGFR expression in RSV infectivity, BEAS-2B cells were first transduced with lentivirus expressing either EGFR-specific shRNA or scrambled (control) shRNA. EGFR shRNA reduced expression of EGFR in BEAS-2B cells by 56% compared to the scrambled shRNA control (Fig 3A and 3B). shRNA knockdown of EGFR in BEAS-2B cells resulted in lower infectivity of A2-2-20F and A2-2-20GF but not A2 (Fig 3C). MeV infection is known to be EGFR-independent; therefore MeV was used as a control for RSV specificity [40]. The knockdown data show EGFR acted as a co-factor contributing to infectivity of RSV expressing the F protein of the clinical isolate 2–20 and suggest RSV F may be interacting with EGFR in a strain-dependent manner.

### RSV F binds EGFR and the extent of binding is RSV strain-specific

**Fig 4:**
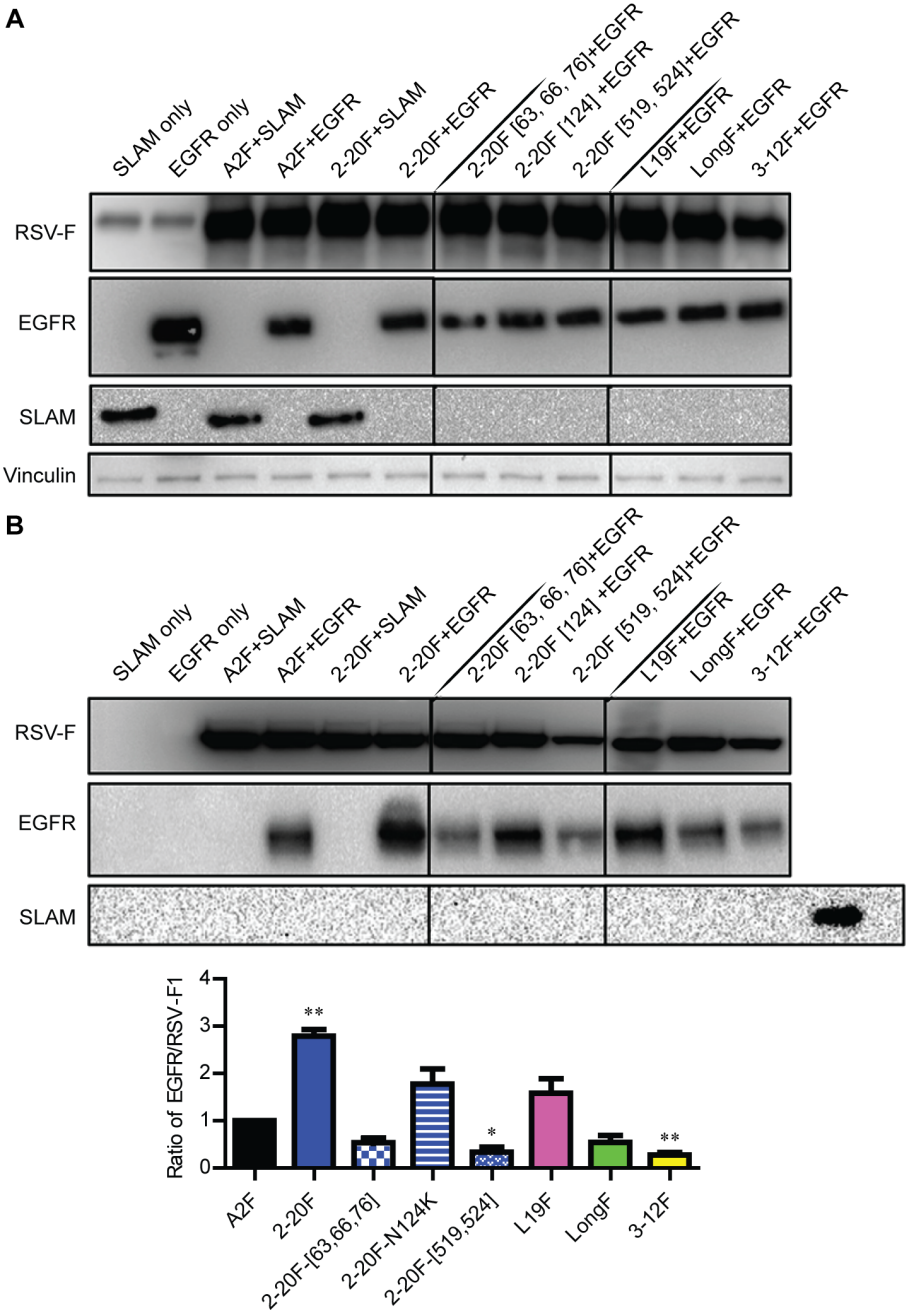
EGFR Interaction with RSV F Depends on Strain-Specificity of F. 293T cells were co-transfected with either SLAM (control) or EGFR and RSV F constructs. Whole cell lysates were obtained after 24 h post-transfection and were either directly blotted (**A**) or immunoprecipitated with motavizumab mAb specific for RSV F (**B**). Whole cell lysates were probed for RSV-F, EGFR, SLAM, and vinculin expression. Proteins immunoprecipitated were probed for RSV-F and EGFR levels. The graph depicts ratios of EGFR to RSV F1 from four separate experiments combined. Error bars represent standard error of the mean (SEM). * and ** represent significant differences (*P* < 0.05 and *P* < 0.005, respectively by ANOVA).

We assessed whether a physical (direct or indirect) interaction can occur between overexpressed RSV F and EGFR. 293T cells were transfected with either EGFR or SLAM and RSV F. All proteins were detected at high levels in whole cell lysates (Fig 4A). RSV F was immunoprecipitated (IP) well with motavizumab mAb and probed for the presence of co-precipitated EGFR. Motavizumab binds an epitope in RSV F that is conserved among RSV strains and between the prefusion and postfusion conformations of the protein [41, 42]. EGFR was detected after precipitation of A2F and 2-20F, and there was a 2.8-fold higher ratio of EGFR bound to 2-20F than to A2F (Fig 4B). We previously deposited the 2–20 genome sequence to GenBank and have modeled the residue differences between A2 F and 2–20 F [27]. To identify putative EGFR interaction sites for RSV 2–20 F, three sets of mutations were introduced into 2–20 F that changed amino acids to the corresponding A2 residues. Mutation sets were introduced into the F head region (T63N, E66K, and G76I triple mutant), stalk region (G519V and K524N double mutant), and in the cleaved 27 residue peptide (pep27) between the furin cleavage sites (N124K) of 2–20 F. Introduction of either the head or the stalk mutation sets resulted in reductions in the efficiency of EGFR-bound to 2–20 F, as detected by IP (Fig 4B). The N124K pep27 mutation did not affect the co-IP efficiency significantly (Fig 4B). Taken together, residues 63/66/76 and residues 519/524 contributed to the co-IP interaction between 2–20 F and EGFR.

We evaluated the Co-IP interaction of F proteins from other RSV strains with EGFR. Strain Line 19 is a laboratory strain that that is more pathogenic in mice, including high viral loads, lung IL-13 levels, and airway mucin expression in BALB/c mice, compared to prototypic strains A2 and Long [43, 44]. RSV strain A2001/3-12 (3–12) is a clinical strain we previously reported to induce lower airway mucin expression in mice than 2–20 [27]. Similar to 2–20, overexpression of line19 F resulted in higher efficiency of EGFR co-IP than did overexpression of A2 F, Long F, or 3–12 F (Fig 4B) The data correlate strength of co-IP F-EGFR interaction *in vitro* with *in vivo* mucogenicity in mice of the strain from which F was derived.

### Co-localization of RSV F and EGFR in cells

**Fig 5:**
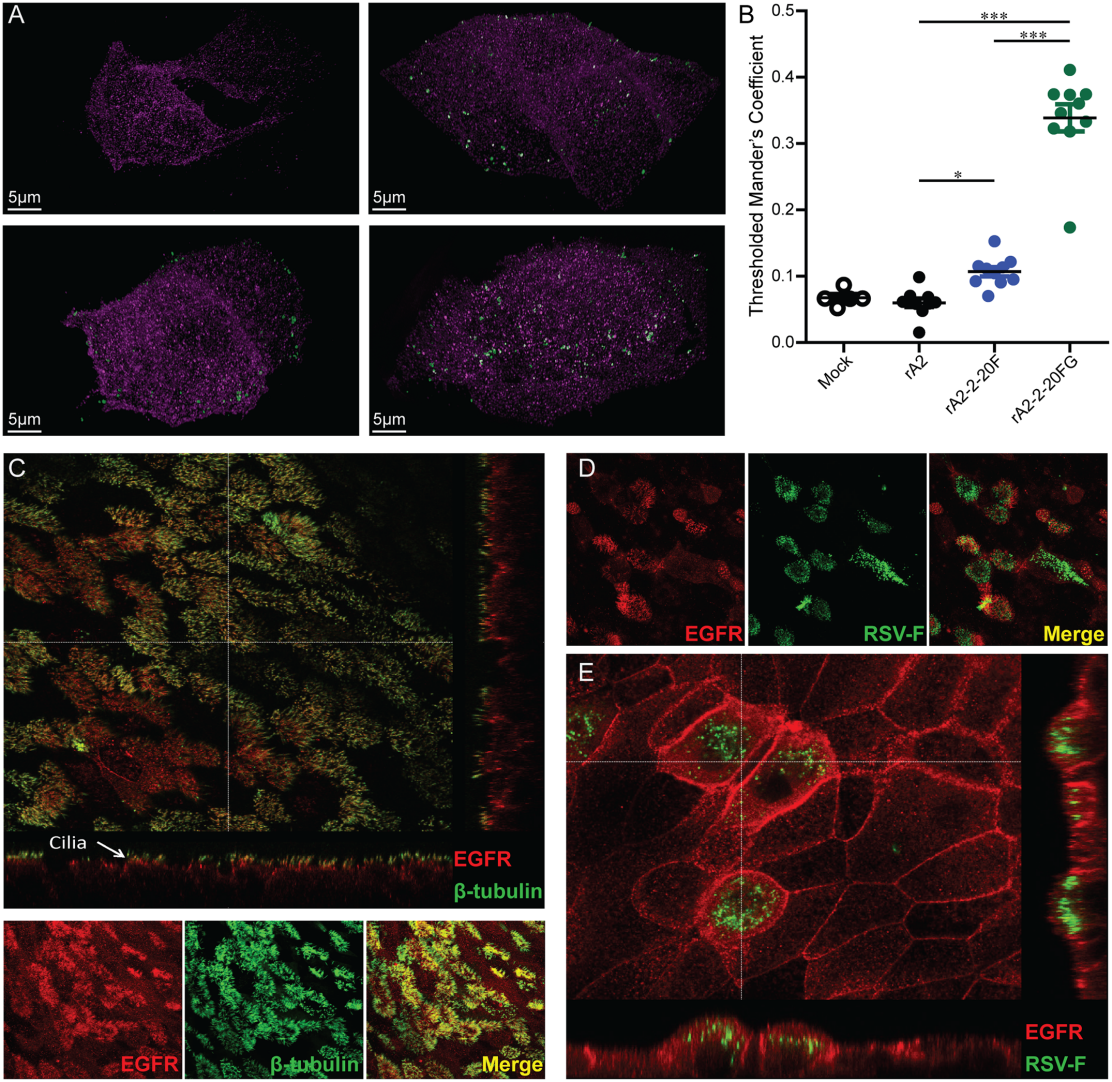
RSV F Co-localizes with EGFR in H292 and WD-PBECs. **(A)** Representative 3D superresolution microscopy images of H292 cells inoculated with A2, A2-2-20F, or A2-2-20GF at 4°C and MOI = 3 then fixed and stained for EGFR and RSV F at the cell surface. Magenta puncta show EGFR staining, green puncta show RSV F staining, and white shows EGFR/F overlap. The scale bar represents 5 μm. (**B**) Quantification of EGFR signal overlap with RSV F signal on the surface of 10 different cells infected with each virus. Error bars represent standard error of the mean (SEM). * and *** represent significant differences (*P* < 0.05 and *P* < 0.0005, respectively by ANOVA). (**C**) Representative WD-PBEC cultures were fixed with PFA and permeabilized before being stained for p-EGFR (red) and beta-tubulin (β-tubulin, green). Images were visualized by confocal microscopy. (**D and E**) WD-PBEC cultures were infected with RSV clinical isolate BT2a and stained for EGFR (red) and RSV F (RSV F, green) expression.

We quantified molecular co-localization between EGFR and RSV F at the cell surface. H292 cells were inoculated with either mock, A2, A2-2-20F, or A2-2-20GF at 4°C, which facilitates attachment but not viral fusion. After one hour and washes, the cells were fixed and stained for RSV F and EGFR then examined by superresolution microscopy at the plasma membrane. There was no significant overlap between EGFR and RSV F puncta in the A2 strain group (Fig 5A and 5B). In contrast, there was significant overlap between RSV F and EGFR puncta on the surface of A2-2-20F- and A2-2-20GF-inoculated cells (Fig 5A and 5B). There were more RSV F puncta per cell in the A2-2-20GF group than the A2-2-20F and A2 groups, consistent with published data that 2–20 G has greater attachment activity than A2 G (Fig 5A) [18]. A2-2-20GF attachment also resulted in greater co-localization of F and EGFR than A2-2-20F (Fig 5A and 5B), suggesting the 2–20 G protein also directs RSV localization at the cell surface. Taken together, there was significant co-localization between 2–20 F and EGFR at the cell surface following virus attachment, which was enhanced by 2–20 G.

To evaluate whether RSV co-localizes with EGFR in a highly relevant primary airway epithelium model, well-differentiated primary pediatric bronchial epithelial cells (WD-PBECs) [45] were analyzed for surface expression of p-EGFR. RSV BT2a induces mucus secretion and goblet cell hyperplasia/metaplasia in WD-PBECs [45]. p-EGFR expression in WD-PBEC cultures was largely focused in the cell membrane on ciliated structures as evidenced by co-localization with beta-tubulin (Fig 5C). WD-PBEC cultures were infected with clinical isolate, BT2a, which has similar kinetics to A2 during infection in HEp-2 cells, but exhibits more cytopathogenesis in WD-PBEC cultures [45]. Many cells infected with clinical isolate BT2a qualitatively co-localized with p-EGFR (Fig 5D). Interestingly, infection of WD-PBECs with BT2a resulted in an apparent increase in surface expression of p-EGFR in RSV-infected cells, but not neighboring non-infected cells (Fig 5E). Collectively, the co-localization data suggest RSV F is closely associated with EGFR at the plasma membrane of H292 and apically in human primary airway epithelial cells.

### Infection of the airway epithelium of mice by RSV A2-2-20F

**Fig 6:**
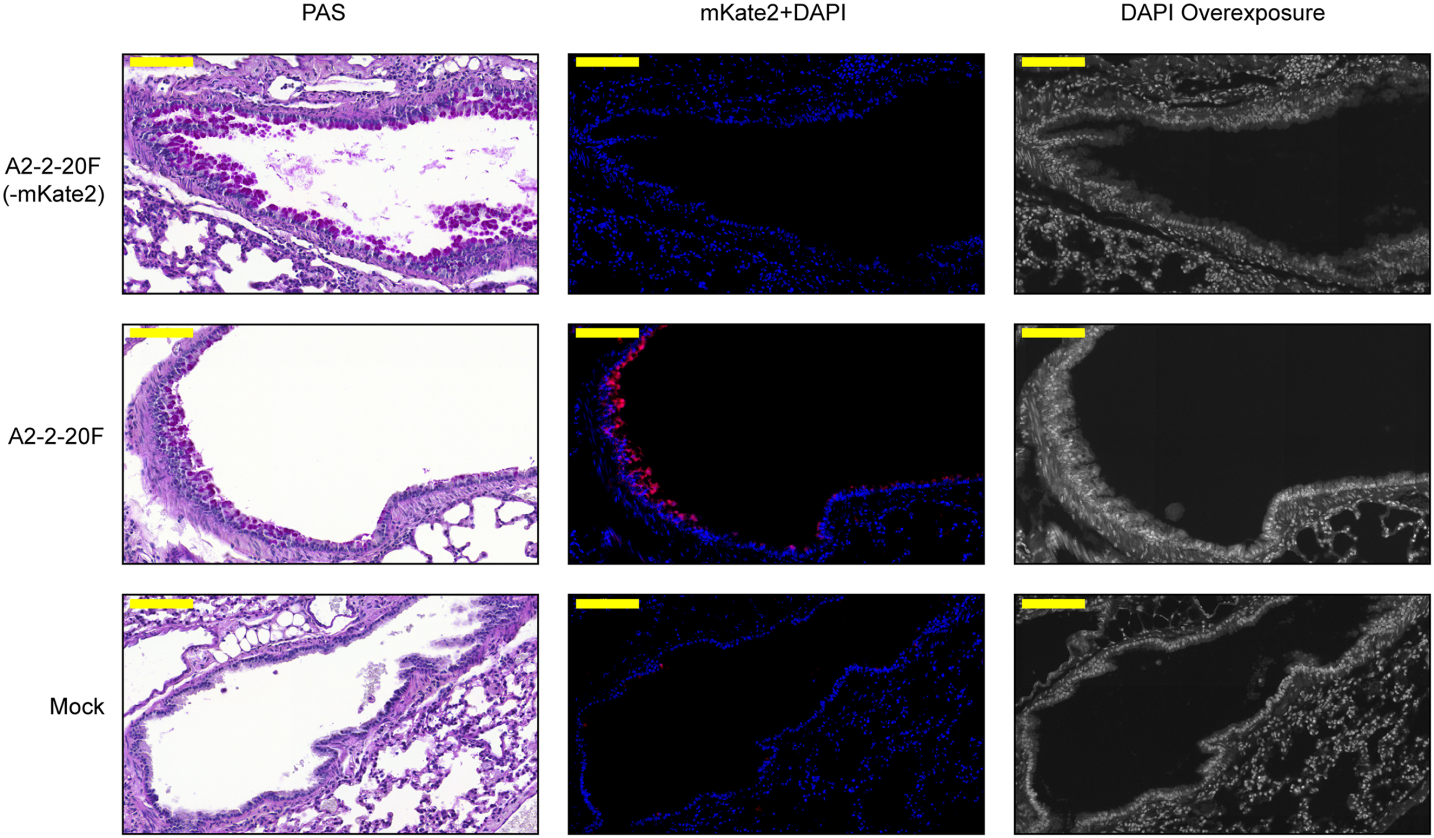
Virally-expressed protein in mucin producing bronchial epithelium. BALB/cJ mice were mock-infected or infected with A2-2-20F (-mKate2) or A2-2-2-20F. Lungs were harvested day 8 post-infection. Serial lung sections were processed for PAS staining or deparaffinized, rehydrated, and DAPI stained. PAS staining shows mucin producing cells (left column). The mKate2 signal was detected by fluorescence microscopy in the adjacent lung section (middle column). A gray level display of the DAPI channel (right column) shows tissue architecture of the mKate2+DAPI section (middle column). Yellow bar represents 100μm.

Infection of BALB/cJ mice with RSV A2-2-20F or the parental isolate 2–20 results in airway mucin expression that peaks approximately day 8 post-infection, a time point when infectious virus is not detectable by plaque assay [27, 28]. We previously demonstrated that 2–20 infects the mouse airway epithelium, detectable by immunofluorescence day one post-infection [27]. We examined whether A2-2-20F also infects the mouse airway epithelium and whether virally expressed proteins can be detected in PAS-positive airways. Mice were mock-infected or infected with A2-2-20F that does expresses mKate2 or A2-2-20F lacking the mKate2-encoding gene. The mKate2 far-red fluorophore has extreme pH stability [46], therefore we hypothesized it will remain functional after histology processing. When adjacent, serial lung sections were compared, mKate2 signal was evident day 8 post-infection in bronchial epithelium that was also producing mucin (Fig 6). To control for autofluorescence, lung sections from recombinant A2-2-2F that does not express mKate2 infected and mock-infected mice were also examined. The mKate2 signal was clearly distinguishable from background (Fig 6), marking cells that were either previously infected and harboring mKate2 or cells actively expressing RSV-encoded gene products. These results correlate A2-2-20F infection with airway mucin expression.

### Inhibition of EGFR ablates RSV A2-2-20F-induced airway mucin expression in mice

**Fig 7:**
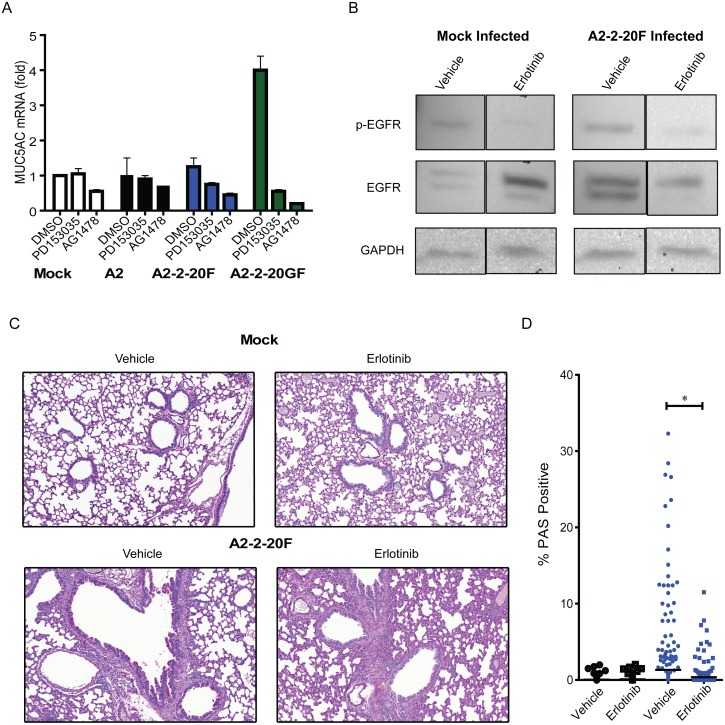
Inhibition of EGFR ablates RSV-induced mucin expression *in vitro* and in mice. **(A)** H292 cells pre-treated with DMSO, 400 nM PD153035, or 10 μM AG1478 were infected with A2 (black), A2-2-20F (blue), or A2-2-20GF (green) at MOI = 1. MUC5AC mRNA levels were quantified by real-time PCR. The fold change relative to mock infection and vehicle-treated cells is shown. (**B—D**) BALB/cJ mice were pre-treated and treated daily with 100 mg/kg erlotinib, an EGFR inhibitor, or vehicle prior to mock infection or 10^6^ PFU A2-2-20F. Lungs were harvested day 8 post-infection. (**B**) Total and P-EGFR levels in lung homogenates. (**C**) Representative lung sections stained with PAS. (**D**) Quantification of the percentage of the area of each airway staining positive in treatment groups. Each symbol represents a single airway and there were at least 285 individual airways from 5 mice shown for each treatment group. One of two replicate experiments with similar results is depicted. * represents a significant difference (*P* < 0.05 by ANOVA) between vehicle and erlotinib treatment.

Muc5ac is a major inducible and secreted mucin protein in the lung that is up-regulated by EGFR activation and during RSV infection [43, 47–49]. We first tested whether RSV infection of these cells results in MUC5AC gene expression. In DMSO-treated serum-starved H292 cells, A2-2-20GF infection resulted in higher MUC5AC mRNA levels than mock, A2, and A2-2-20F infection (Fig 7A). Therefore H292 cells provide an *in vitro* model of RSV strain-specific induction of mucin expression. Serum-starved H292 cells were treated with EGFR pathway inhibitors PD153035 and AG1478, mock-infected or infected with RSV strains A2, A2-2-20F, and A2-2-20GF, and MUC5AC expression was quantified. In the presence of either inhibitor, MUC5AC mRNA fold-change in A2-2-20GF infected cells was ablated. The combination of RSV 2–20 F and 2–20 G was important for MUC5AC induction in this *in vitro* model, consistent with the efficient attachment function of 2–20 G (Fig 5A and ref [18]), and the EGFR pathway was critical for MUC5AC induction.

We tested the role of EGFR signaling in A2-2-20F-induced airway mucin expression. BALB/cJ mice were pre-treated with erlotinib, a specific quinazoline derivative that binds to and inhibits EGFR tyrosine kinase activity, or vehicle suspension, and tested for suppression of EGFR activation in mouse lung homogenates [50]. Erlotinib caused a reduction in the signal of p-EGFR in lungs from both mock-infected and A2-2-20F-infected mice (Fig 7B). Mice pre-treated then treated daily with vehicle or erlotinib were infected with mock or A2-2-20F. Lungs were harvested day 8 post-infection and processed for periodic acid-Schiff (PAS) stains of goblet cell hyperplasia/metaplasia (Supplemental Fig 2), a surrogate of airway for mucin expression. Our lab uses a digital pathology system to morophometrically quantify PAS positivity in all airways involved in lung sections [27, 28]. Mice treated with erlotinib and infected with A2-2-20F had significantly less airway goblet cell hyperplasia/metaplasia than vehicle-treated, A2-2-20F-infected mice (Fig 7C and 7D). Taken together, these data identify a novel role for EGFR signaling in mediating RSV-induced mucin expression and airway pathology.

## Discussion

RSV disease in infants is associated with airway obstruction, lung inflammation, epithelial cell sloughing, and mucus production [51, 52]. The relative contributions of sloughed epithelium and mucus production to airway obstruction remain unknown [53, 54]. Distal airways are thought to have fewer mucin-secreting cells than larger airways, but overabundant mucus production is associated with infant bronchiolitis clinically [53–55]. The clinical isolate strain 2–20 to a degree recapitulates human RSV disease in the BALB/c mouse model of RSV infection [27, 28, 56]. The prototypical A2 strain does not cause airway mucin expression in mice, and chimeric RSV strains A2-2-20F is mucogenic like parental 2–20, implicating the F protein in mucin induction *in vivo* [28, 44]. RSV 2–20 has somewhat altered tropism in the mouse because it infects the airway epithelium and alveolar epithelial cells, whereas A2 infects predominantly alveolar epithelial cells [27]. 2–20 and A2-2-20F cause more airway necrosis in mice than A2 [28]. Therefore, infection of the mouse airways correlates with mucin induction in this model. Here, we investigated mechanisms of 2–20 F-induced airway mucin induction. In cultured cells, 2–20 F exhibited greater functional and physical interaction with EGFR than A2 F, and EGFR was able to enhance the fusion activity of the 2–20 F protein. Chemical inhibition of EGFR signaling reduced infectivity of 2–20 F-expressing RSV and ablated mucin induction *in vitro* and *in vivo*. We report that RSV F interacts with and activates EGFR and that EGFR contributes to infection *in vitro* and plays a critical role in RSV-induced mucin expression.

Our study shows F and EGFR interact functionally and physically. Expression of the 2–20 F protein potently activated EGFR, as measured by p-EGFR levels in cells. The efficiency of co-IP of EGFR with F depended on strain specificity of the expressed F protein. EGFR had the highest co-IP efficiency with 2–20 and line 19 F proteins, strains previously shown to be mucogenic in mice [27, 28, 44]. We mapped the enhanced co-IP efficiency with 2–20 F to two domain differing between 2–20 F and A2 F, residues 63/66/76 and residues 519/524. Amino acids 63, 66, and 76 cluster at the top of the prefusion F trimer. This region overlaps prefusion-specific antigenic site ∅, and the adjacent residue 67 is important for RSV F prefusion stability [11, 57]. As we discussed in Stokes et al, residues 519 and 524 in RSV F are membrane-proximal in the stalk, a region implicated in regulating Hendra virus F protein triggering via stabilization of the pre-fusion form [28, 58]. In our co-IP experiments, we expressed functional F, which for RSV does not require triggering by the attachment protein, so prefusion and postfusion forms were present. We speculate that 63/66/76 and 519/524 regions regulate prefusion stability, which may relate to EGFR co-IP efficiency between F species and mutants. In the DSP fusion assay, EGFR boosted 2–20 activity, so we predict the functional interaction occurs prior to postfusion F formation. Additional studies and reagents will be required to further elucidate F-EGFR molecular interactions.

RSV infection was previously shown to activate EGFR. The A2 strain activates EGFR in cells, resulting in delayed apoptosis by ERK activation and production of the pro-inflammatory cytokine IL-8 production [24]. RSV A2 strain was shown to activate EGFR following virus attachment, leading to macropinocytotic endocytosis [26]. In that study, EGFR depletion in HeLa cells by siRNA delivery resulted in reduced infectivity of the A2 strain, whereas we found that EGFR depletion in BEAS-2B cells reduced infectivity of RSV expressing 2–20 F but not of the A2 strain. The discordant findings may be related to the cell line and/or the efficiency of EGFR knockdown. Recently, RSV activation of EGFR, in addition to influenza A (H1N1) and rhinovirus infections, led to suppression of IRF1-dependent CXCL10 production [25]. CXCL10 is expressed in airway epithelial cells, is a ligand of CXCR3 (a key regulator of leukocyte trafficking), and when elevated is associated with obstructive airway diseases [25, 45, 59, 60]. Using RSV 2–20 F-expressing viruses in future studies may shed additional light on entry mechanisms, such as macropinocytosis, and downstream immunopathogenesis such as IL-8 expression, neutrophil recruitment, and CXCL10 expression.

**Fig 8:**
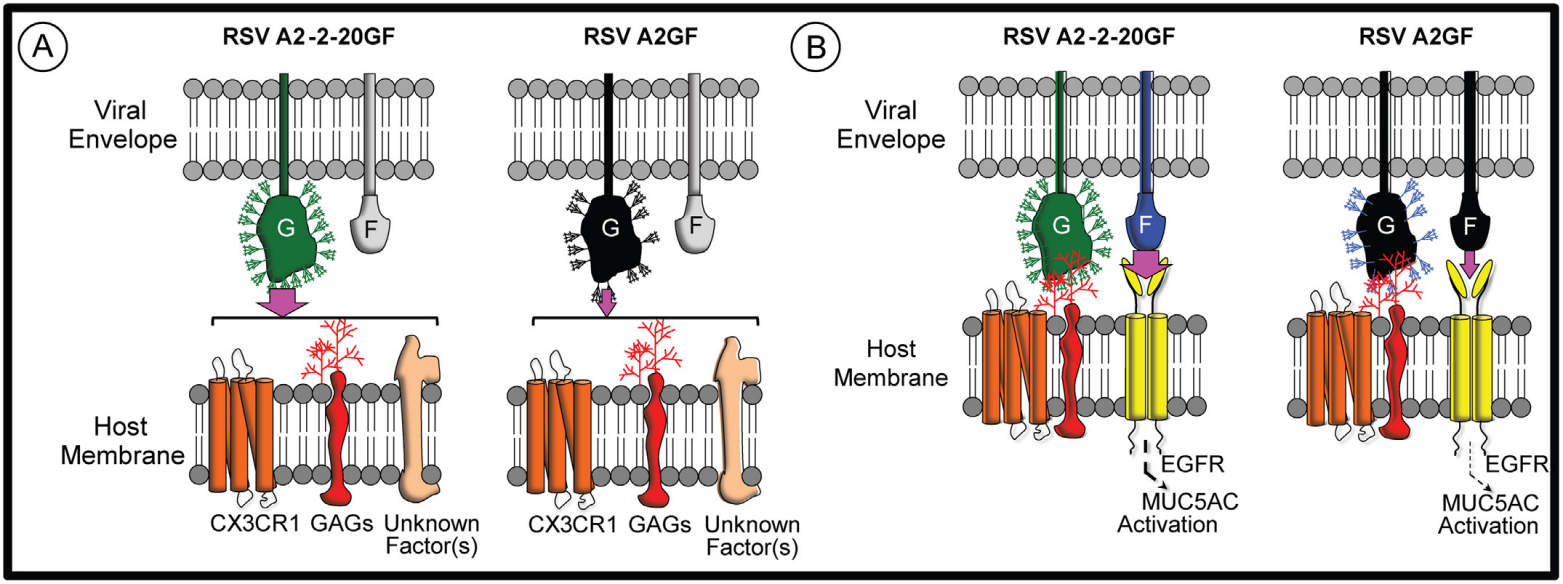
Model of RSV G and F interactions with host factors at the cell surface. **(A)** RSV G mediates attachment by interacting with GAGs, CX3CR1, and potentially other factor(s). RSV 2–20 G is more heavily glycosylated and confers greater cell attachment activity than A2 G, as indicated by the width of the purple arrows. **(B)** RSV F interacts with EGFR, and RSV 2–20 F does so more strongly than A2 F (purple arrows). The F-EGFR interaction contributes to infection, EGFR activation, and induction of mucin gene expression.

The capacity of RSV F to engage EGFR during infection may depend in part on a function of the RSV G protein. We observed an appreciable increase in MUC5AC expression in H292 cells when using strain A2-2-20GF, not A2 or A2-2-20F. In these cells, 2–20 G conferred greater attachment, as measured by superresolution microscopy, consistent with the sequence-based prediction that 2–20 G has more glycosylation sites and our recent published data that 2–20 G has a higher apparent molecular weight than A2 G and confers enhanced cell attachment in vitro [18]. The co-localization quantification in H292 cells revealed that, irrespective of the abundance of RSV F puncta, 2–20 G greatly enhanced signal overlap between F and EGFR, suggesting 2–20 G alters location of RSV on the plasma membrane. Our current working model is that initial 2–20 G interactions with CX3CR1, GAGs, and/or other factors mediates attachment that likely precedes F-EGFR interaction, EGFR activation, and infection (Fig 8). Further studies will need to evaluate the role of G in F-EGFR interactions during infection in cells and animal models.

In summary, we for the first time identified a host protein that both interacts with the RSV F protein and promotes fusion. EGFR was expressed at the apical surface of differentiated pediatric bronchial epithelial cells, and RSV F and EGFR co-localized in infected cells. Using clinically relevant RSV strains and infection models, we found that EGFR is critical for RSV-induced airway mucin expression and laid the groundwork for defining the molecular interaction between F and EGFR.

## Materials and Methods

### Ethics statement

All animal procedures were conducted according to the guidelines of the Emory University Institutional Animal Care and Use Committee, under approved protocol number 2001533. The study was carried out in accordance with recommendations in the Guide for Care and Use of Laboratory Animals of the National Institute of Health, as well as local, state, and federal laws.

### Cells and reagents

The media components and origins of HEp-2, BEAS-2B, 293T, and BSR-T7/5 cells we use are previously described [27, 28]. NCI-H292 cells were purchased from ATCC (CRL-1848) and propagated in RMPI-1640 supplemented with 10% FBS (Hyclone, Thermo Scientific), 0.01 M HEPES, and 25 mM D-glucose. Normal human bronchial epithelial (NHBE) cells were obtained from Lonza (Allendale, NJ) and differentiated on collagen-coated 24-well transwell supports at air-liquid interface as we described [38]. The human codon bias-optimized RSV A2 F and 2–20 F expression plasmids are described, and the RSV 2-20GF-expression plasmid was generated using the same strategy [28]. EGFR cDNA in a pBABE retroviral vector was transferred into pTriEx-3 using standard restriction enzyme cloning to yield pTriEx-3-EGFR, and the sequence of EGFR was confirmed. A full-length SLAM cDNA was extracted from Vero-SLAM cells and cloned into the pCG expression plasmid. DSP_1-7_ and DSP_8-11_ plasmids were provided by Naoyuki Kondo and Zene Matsuda [61]. Lipofectamine 2000 (Life Technologies) was used according to the provided protocol for all cell transfections, with the exception that DNA/liposome mixture remained on cells overnight for RSV rescue. The anti-RSV F mAb motavizumab was generously provided by Nancy Ulbrandt (MedImmune AZ). Mouse anti-SLAM was purchased from Abcam (clone ab2604) and mouse anti-vinculin was purchased from Fisher Scientific (clone VLN01). HRP-conjugated secondary antibodies for immunoblotting were purchased from Jackson Immunoresearch. Compound AG1478 (LC Laboratories) was diluted in DMSO. Recombinant human EGR was obtained from Lonza (Cat #00556827).

### Viruses

We previously reported generation of recombinant RSV encoding the far-red fluorescent protein monomeric Katushka-2 (mKate2) in the first gene position [62]. This virus was recovered on BSR-T7/5 cells transfected with pSynkRSV-line19F BAC together with helper plasmids encoding codon-optimized N, P, L, and M2-1 [62]. The F gene of BAC pSynkRSV-line19F, flanked by *Sac*II and *Sal*I sites, was replaced with a synthetic cDNA (GeneArt, Life Technologies) encoding either the A2 strain F open reading frame, the 2–20 strain F open reading frame, or the 2–20 strain G and F open reading frames, flanked by non-coding regions identical to those in pSynkRSV-line19F BAC [18]. The recombinant A2-2-20F that does not encode mKate2 (A2-2-20F –mKate2) was previously described [28]. The kRSV-A2, kRSVA2-2-20F, and kRSVA2-2-20GF viruses were plaque-purified and amplified in HEp-2 cells. Virus stocks used were sequence confirmed for the G and F genes and determined to be *Mycoplasma* free using the Venor GeM *Mycoplasma* detection kit (Sigma-Aldrich). RSV clinical isolate BT2a was used for infection of well-differentiated primary pediatric bronchial epithelial cells (described below) [45]. Measles virus expressing GFP (MeV-GFP) used in this study was previously described [63].

### EGFR knockdown

shRNA constructs targeting EGFR (TRCN0000039635, TRCN0000039634, TRCN0000039633, TRCN0000010329, TRCN0000121067) were purchased from Sigma. The control plasmid pLKO.1, which has a scrambled shRNA, was also purchased from Sigma. Two lentivirus helper plasmids, psPAX2, and pMD2.VSVG, were kindly provided by Gregory Melikian (Emory University). Lentiviruses for the puromycin pLKO constructs were produced in 293T cells with two helper constructs. 293T cells (6.5 × 10^5^) were transfected with pLKO containing the EGFR shRNA, psPAX2, and pMD2.VSVG. As a control, 293T cells were transfected with pLKO.1 containing a scramble shRNA. Supernatants were harvested at 24 h following transfection. For infection, 30 to 40% confluent BEAS-2B cells were spinoculated with virus-containing supernatant at 4°C, 2900 x g. Following overnight incubation, media containing 1 μg/mL puromycin was added for 48 hours post-infection. A kill curve of puromycin on BEAS-2B cells determined that 1μg/mL puromycin killed 100% of cells. Surviving BEAS-2B cells following puromycin treatment were used for RSV infection and subsequently used for determination of EGFR knockdown efficiency.

### Flow cytometry

Flow cytometry analysis was performed to quantify RSV infectivity and levels of EGFR surface expression. Phycoerythrin-EGFR antibody (sc-101 PE, Santa Cruz) was used for the detection of EGFR on non-permeabilized 293T or BEAS-2B cells using an LSRII flow cytometer (BD Biosciences). Motavizumab and Alexa 488-anti-human secondary Ab (H17101, Life Technologies) were used for measuring RSV F cell surface expression on non-permeabilized 293T cells. BEAS-2B cells were harvested, fixed, and acquired using a 561nm laser. Isotype control antibody (PE-IgG) was used as a negative control. For measuring of RSV and MeV infectivity, cells were harvested 24 hr post-infection and acquired using an LSRII, by detecting the mKate2 and GFP signals, respectively. Data were analyzed using FlowJo software (TreeStar, Ashland, OR).

### RSV F protein co-ip and western blots

293T cells transfected with equal molar amounts of DNA were used for each group, and plasmid quality was checked via agarose gel and spectrophotometer quantitation. 24 h after treatment, cells were harvested. Cell pellets were thawed on ice and lysed with RIPA buffer (Sigma) plus protease inhibitors (Thermo Scientific). Protein concentrations were determined using Bradford Reagent (Sigma). 50% (v/v) protein-A magnetic beads (Cell Signal) were conjugated with motavizumab by mixing 50 μL of bead slurry with 500 μL ice cold PBS and 0.75 μg of motavizumab (1.75 mg/mL) in a microcentrifuge tube rotating end-over-end for 4 h at 4°C. Excess antibody was washed from the beads by pelleting, aspirating the antibody suspension, and washing 3 times with 1 mL of RIPA buffer. 120 μL of cell lysate was pre-cleared with 30 μL of non-conjugated bead slurry. 100 μL of cleared lysate was then added to the conjugated bead pellet with 10 μL of 10% BSA (Sigma). The lysate/bead slurry was allowed to mix overnight, rotating end over end at 4°C. Beads were removed using a magnetic tube rack on ice at 4°C, were washed 4x in ice cold RIPA, and washed 1x in ice cold PBS. Beads were then pelleted by and re-suspended in 3x SDS loading buffer. For Westerns, lysates or beads mixed with 3x SDS loading buffer were heated 95°C 5 min then fractionated on 10% SDS-Page gels (Bio-Rad). Proteins were transferred to PVDF membranes (Bio-Rad). Membranes were blocked with 2% non-fat milk, 1% FBS (Gemini) in TBST. Membranes were incubated in primary antibody overnight (p-EGFR, EGFR, or SLAM) or 2 h (motavizumab or vinculin).

### Fusion assay

The dual-split protein (DSP) reporter cell-cell fusion assay was previously adapted to measuring RSV F protein activity [28, 33, 34]. 293T “effector” (cis) cells were transfected with RSV A2 F or 2–20 F and DSP_1-7_ in the presence of fusion inhibitor BMS-433771 (a gift from Jin Hong, Alios Biopharma, San Francisco, CA). 293T “target cells” (trans) were transfected with DSP_8-11_ and pCG-SLAM or pcDNA3.1 empty vector. Effector cis or target trans cells were transfected with pTriex3-EGFR. Effector and target cells were washed with PBS 24 h post-transfection and harvested by pipetting in media containing EnduRen live cell luciferase substrate (Promega). Equal volumes of effector and target cells were mixed and placed into an opaque 96-well plate in quadruplicate. Plates were incubated at 37°C and luciferase activity as a measure of cell-cell fusion was assayed on a TopCount Luminescence counter (Perkin Elmer) 4, 6, and 8 h after cell mixing. A positive control of DSP_1-7_ and DSP_8–11_ transfected into the same cell population was used to validate replicates.

### Real-time PCR

H292 cell monolayers were serum-starved for 24 h then mock-infected or infected with RSV. The cells were washed with PBS and lysed with TRIzol reagent (Life Technologies) at 20 h post-infection. Total RNA was isolated according to the TRIzol protocol. Quantitative real-time PCR was performed using the AgPath-ID OneStep RT-PCR kit (Applied Biosystems) and an ABI 7500 sequence detector system (Applied Biosystems). The primers and probes for MUC5AC gene (forward, 5′CGTGTTGTCACCGAGAACGT3′; reverse, 5′ ATCTTGATGGCCTTGGAGCA 3′, probe, 5′ Fam- CTGCGGCACCACAGGGACCA-BHQ-1 3′) were obtained from Integrated DNA technologies (IDT) [64]. The primers and probes for GAPDH, the control, were forward, 5’ GAAGGTGAAGGTCGGAGT 3’, reverse, 5’ GAAGATGGTGATGGGATTTC 3’, and probe, 5’ Fam CAAGCTTCCCGTTCTCAGCC 3’. Threshold cycles (Ct) and ΔCt for each sample was calculated. Assays were performed in duplicate in 3 independent experiments.

### Immunofluorescence in H292 and WD-PBEC cells

H292 cells were cultured on 35-mm glass-bottom dishes (MatTek Corp). Cells were inoculated at MOI = 3 at 4°C rocking for 1 h., conditions at which attachment but not fusion can occur, washed twice in chilled PBS, and fixed in 10% buffered formalin (Thermo-Fisher) for 10 min. Cells were then washed 3X at room temperature with PBS. The fixed cells were blocked overnight in serum-free protein block (Dako). Antibodies were diluted in an antibody diluent with background reducing components (Dako). For the detection of RSV-F, a 1:2,000 dilution of 1.75 mg/mL motiavizmab was utilized. EGFR was detected with a rabbit polyclonal (Millipore) used at a 1:500 dilution. Primary antibodies were allowed to bind to cells for 4 h rocking at 4°C. Secondary antibodies anti-human IgG, IgA FITC conjugated (LifeTech) and anti-rabbit Alexa-568 (LifeTech) were used at dilutions 1:5,000 and 1:2,000 respectively. Secondary antibodies were incubated for 1 h rocking at room temp, followed additional 3 wash steps. Cells were kept at 4°C under PBS until imaging. Super-resolution images were acquired using a DeltaVision OMX Blaze (GE Healthcare Life Sciences) for three-dimensional structured illumination microscopy (3D-SIM). 3D-SIM reconstructions were generated by softWoRx (v6.1.3). The reconstructed files were further analyzed in Imaris (v8.1.2) where appropriate channel thresholds were manually set. An overlap mask channel was created using Imaris where the thresholded Mander's coefficient was calculated to quantify 3D overlap.

For WD-PBECs, pediatric bronchial epithelial cells (PBEC) were obtained, via written parental consent, from bronchial brushings of children undergoing elective surgery at the Royal Belfast Hospital for Sick Children, and the procedures were approved by the Office of Research Ethics Committees Northern Ireland [45]. PBEC were expanded in collagen-coated flasks using airway epithelial cell media and supplements (Lonza), then seeded onto transwell inserts (Corning), and then air-liquid interface (ALI) cultures were initiated and maintained 21 days in order to establish well-differentiated (WD)-PBECs, as described in further detail [45]. Paraformaldehyde-fixed and permeabilized WD-PBEC were stained for anti-β-tubulin, MUC5AC, or RSV F protein expression as described [45] and were stained with anti-phospho-(p)-EGFR (Abcam, ab40815). WD-PBEC cultures were infected with RSV subgroup A clinical isolate BT2a as described [45]. Fluorescent images were obtained with a SP5 confocal DMI 6000 inverted microscope (Leica).

### Erlotinib treatment of mice and quantification of airway mucin expression

7-week old female BALB/cJ mice (The Jackson Laboratory) were orally gavaged with 100 mg/kg of erlotinib (Selleck Chemicals LLS, Catalog S1023) or vehicle (0.5% carboxymethylcellulose /0.1% Tween 80) in a total volume of 100 μL daily, beginning two days prior to infection, and continuing for the duration of the experiment. Mice were infected intranasally with 1 x 10^6^ PFU of A2-2-20F or mock virus preparation. On day 8 post-infection, the lungs were harvested and placed in 10% neutral buffered formalin for histopathology sectioning and periodic acid Schiff (PAS) staining for goblet cell hyperplasia as a measure of airway mucin expression. PAS positivity was quantified for greater than 285 individual airways total from 5 separate mice per group by digital morphometric analysis as described previously [27]. In a separate group of mice, the left lung was harvested, snap frozen, and later homogenized in RIPA buffer containing a protease inhibitor cocktail. The amount of total protein the lung homogenates was determined by Bradford assay and equivalent proteins from each group were loaded on a 4–17% SDS-PAGE gel and separated by electrophoresis before Western blotting for total EGFR (Abcam, AB15669) or p-EGFR Monoclonal (Abcam, AB24928).

### Fluorescent imaging of serial lung sections

7-week old female BALB/cJ mice (The Jackson Laboratory) were infected intranasally with 1 x 10^6^ PFU of A2-2-20F (-mKate2), 1 x 10^5^ FFU of A2-2-2-20F, or mock virus preparation. On day 8 post infection, lungs were harvested and prepared as above for PAS staining or, after sectioning, were deparaffinized with Clear-rite (Thermo Scientific), rehydrated through graded alcohols to water, and then stained with Prolong DAPI Gold (Life Technologies). PAS slides were imaged using a Mirax Imaging System as descried previously [27]. DAPI-stained slides analyzed by fluorescence microscopy using the Mirax Image System. Equal exposure time was used for each channel (DAPI 120μs, mKate2 900μs) across all groups. Excitation was provided by HXP-120 light source (LEj) through Zeiss filter set 2 (DAPI) or 45 (mKate2). Images were analyzed in Panoramic Viewer v1.15.2 (3DHISTECH), where pseudocoloring levels were kept constant for each channel across all groups. Images of representative airways from the central portion of each lung were exported as TIF files.

## Supporting Information

S1 Fig F and EGFR cell surface expression. 293T cells were transfected with EGFR and either A2 F or 2–20 F. (**A &B**) Cell were harvested and (non-permeabilized) probed with mAbs to either EGFR (**A**) or RSV F (**B**) and analyzed by flow cytometry. (**C**) The quantification of three experiments combined showing that EGFR and RSV F surface expression do not vary between cells transfected with EGFR and A2 F or EGFR and 2–20.
(TIF)

S2 Fig Goblet cell hyperplasia/metaplasia resulting from RSV A2-2-20F infection. Lungs from vehicle-treated and mock-infected (A-B) or RSV A2-2-20F-infected (C-D) mice were harvested at day 8 p.i., serially sectioned, and stained with hematoxylin and eosin (H&E) (A and C) or PAS (B and D). Arrows indicate goblet cells (C) and mucin (D). Magnification, 70 ×.
(TIF)
