# Supplementary figures and images for: EGFR Interacts with the Fusion Protein of Respiratory Syncytial Virus Strain 2-20 and Mediates Infection and Mucin Expression
Source: PLoS Pathog. 2016 May 6;12(5):e1005622. doi: 10.1371/journal.ppat.1005622 (PMC4859522; doi:10.1371/journal.ppat.1005622)

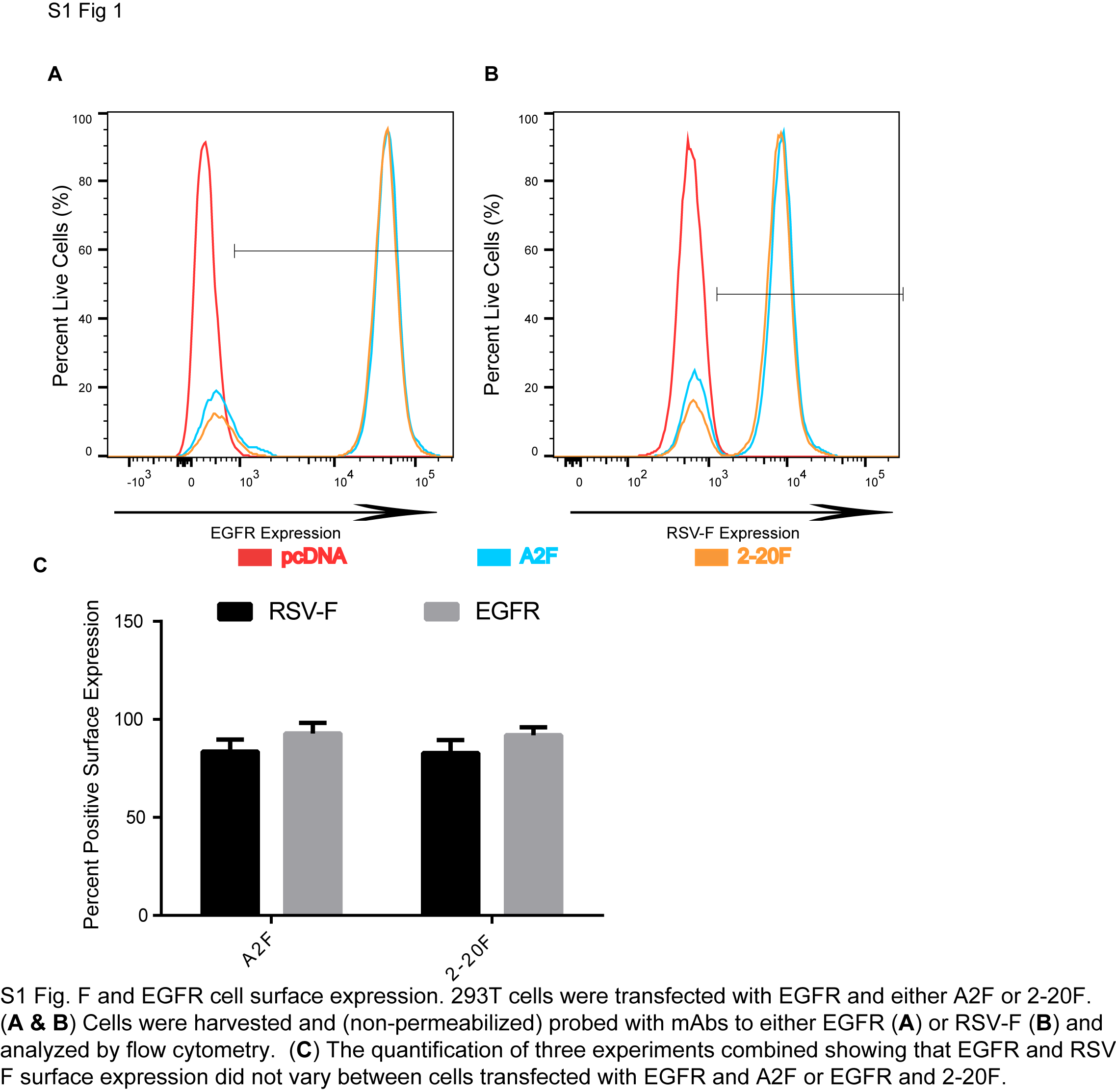

Supplement: S1 Fig — 293T cells were transfected with EGFR and either A2 F or 2–20 F. (A &B) Cell were harvested and (non-permeabilized) probed with mAbs to either EGFR (A) or RSV F (B) and analyzed by flow cytometry. (C) The quantification of three experiments combined showing that EGFR and RSV F surface expression do not vary between cells transfected with EGFR and A2 F or EGFR and 2–20. (TIF) [file ppat.1005622.s001.tif]

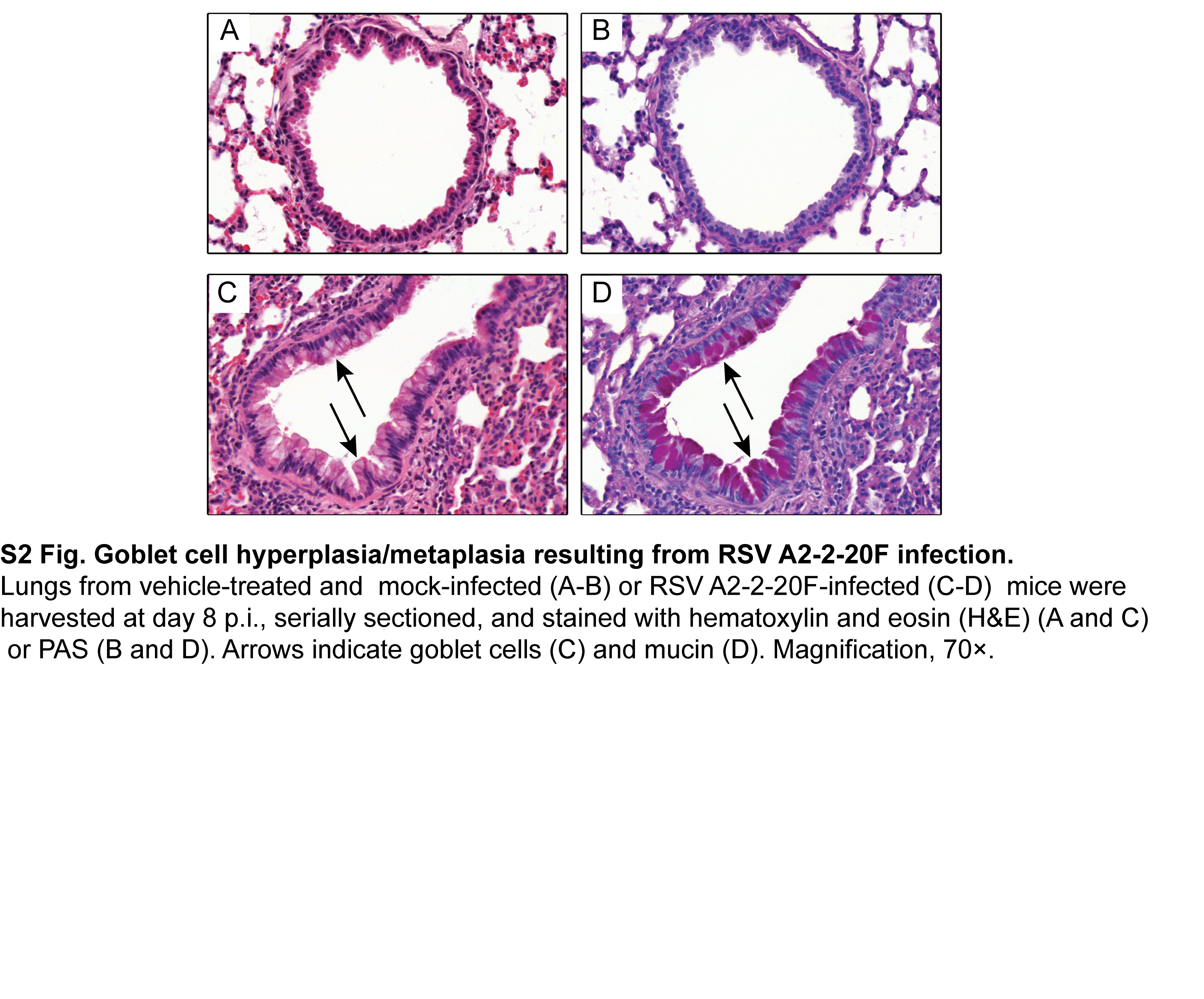

Supplement: S2 Fig — Lungs from vehicle-treated and mock-infected (A-B) or RSV A2-2-20F-infected (C-D) mice were harvested at day 8 p.i., serially sectioned, and stained with hematoxylin and eosin (H&E) (A and C) or PAS (B and D). Arrows indicate goblet cells (C) and mucin (D). Magnification, 70 ×. (TIF) [file ppat.1005622.s002.tif]
